# Objective Evaluation of a Simulation Course for Residents in the Pediatric Emergency Medicine Department: Breaking Bad News

**DOI:** 10.7759/cureus.3903

**Published:** 2019-01-16

**Authors:** Yih Ying Yuan, Susan Scott, Ngoc Van Horn, Oluwaseun Oke, Pamela Okada

**Affiliations:** 1 Pediatric Emergency Medicine, University of Texas Southwestern, Dallas, USA; 2 Miscellaneous, Chidlren's Health System of Texas, Dallas, USA

**Keywords:** breaking bad news, simulation, medical education, pediatrics emergency medicine

## Abstract

Introduction: Breaking bad news (BBN), especially in the pediatric emergency medicine department, requires significant skill and delicacy due to the acute context of a busy emergency department (ED) and the lack of prior rapport with the patients and families. Pediatric literature on breaking bad news has mostly focused on pediatric oncology and pediatric critical care, with limited literature focused on pediatric emergency medicine. Review of the literature also reveals that most existing studies solely assess the learners’ self-ratings of efficacy and comfort, and far fewer studies objectively evaluate learners’ actual performance using simulation. Our objectives for this study was to use an objective assessment tool to assess residents’ breaking bad news skills, pre- and post-simulation training, specifically in the setting of a pediatric emergency medicine department.

Methods: 34 residents were evaluated on their performance in breaking bad news via videotaped simulation encounters before and after teaching intervention. The "Modified Breaking Bad News Assessment Scale” (mBAS) was used as the assessment tool. A paired t-test analysis was conducted to examine the mean difference in pre- and post-simulation scores in each of the five mBAS domains.

Results: Breaking bad news performance score improves one to two weeks post-intervention, and was statistically significant in three of five domains.

Conclusion: Our study shows that breaking bad news is a teachable skill that can be improved by simulated education in the pediatric emergency medicine department. This study demonstrates the utility of simulation course in improving breaking bad news skills in the pediatric emergency medicine department. Future work in developing focused simulation curriculums is important to improve provider communication skills and patient-physician relationships.

## Introduction

Breaking bad news (BBN), especially in the pediatric emergency medicine department, requires significant skill and delicacy due to the acute context of a busy emergency department (ED) and the lack of prior rapport with the patients and families [1]. There are no typical approaches physicians use to deliver bad news [2], which may range from new diagnosis of chronic or terminal illness to death notification [1]. However, the way in which bad news is delivered not only greatly impacts the recipients’ psychological adjustment to the news, but the emotional effect also is difficult to amend if the news was delivered poorly [3-7]. In contrast, good physician-patient communication improves patients’ and families’ emotional health and treatment outcomes [3-8]. Research also indicates that BBN can lead to anxiety, emotional exhaustion and burnout, and physicians are often underprepared due to inadequate training and lack of supervisor support [9-10].

Despite that communication skills are one of the six core competencies, according to the Accreditation Council for Graduate Medical Education (ACGME) [11-14], few training programs offer the curriculum to enhance this education [15-18]. In addition, many trainees learn from direct observation [9], but this education varies according to senior physicians’ skills, and many trainees report they often lack training, supervision, and feedback after BBN to patients [8].

Simulation has long been used to teach trainees procedures and communication skills, and BBN training has become a recent area of interest in simulation [19]. This idea originated from the field of oncology, where research showed poor skills of providers in BBN, leading to patient dissatisfaction and provider stress [19]. Simulation enhances residents’ comfort in BBN, which translate into more effective communication skills throughout their training [15]. This skill is particularly important in the pediatric emergency medicine department due to the acuity and unexpected adverse events that increase physician stress and patient dissatisfaction.

Pediatric literature on BBN has mostly focused on pediatric oncology and pediatric critical care, with limited literature focused on pediatric emergency medicine [20]. Review of literature also reveals that most existing studies solely assess the learners’ self-ratings of efficacy and comfort, and far fewer studies objectively evaluate learners’ actual performance using simulation [21]. Our aim for this study was to use an objective assessment tool to assess residents’ BBN skills, pre- and post-simulation training, specifically in the setting of a pediatric emergency medicine department.

## Materials and methods

Participants 

Pediatric, emergency medicine and family medicine residents rotating through the ED in our institution, a free-standing tertiary children’s hospital and an academic center, were invited to participate. The study period was from July 2017 to November 2017. There were no specific selection criteria, and residents participated based on their availability and willingness. The only exclusion criteria included residents who had greater than five hours of prior formal didactic training in BBN. Institutional Review Board (IRB) and site approval from our hospital were obtained prior to the recruitment of trainees.

Rationale of the assessment tool selected

The objective assessment tool selected for this study was the "Modified Breaking Bad News Assessment Scale” (mBAS) (Appendix 1), originally designed and published by Miller et al. [22], in the British Journal of Cancer, as a collaboration between the oncology and psychiatry departments, of the University of Oxford, UK. The scale was later validated in a BBN study on medical students in 2012 [23]. It is a detailed and structured checklist that contained 22 items distributed over five different domains of communication behaviors relevant to BBN. For ease of scoring, the questions are grouped by sections into the chronological order one would expect in an actual patient encounter [22-23]. The five domains include A: setting the scene, B: breaking bad news, C: eliciting concerns, D: providing information, and E: general considerations. These items are rated on a Likert scale of one (very good) to five (very poor). The mBAS was selected for several reasons. First, it was shown to generate scores for each category listed above, and therefore helped identify strengths and weaknesses in individual performance. Second, it standardized scores for overall performance and demonstrated good utility in that raters required minimal expertise in the field and minimal training to complete the mBAS. Third, there is limited availability of validated tools to objectively evaluate performance on BBN. 

The simulation cases

The Principal Investigator (PI) created two simulation cases to be randomly assigned in the study. Case one involves a one-year-old who presents with viral symptoms found to have new-onset leukemia. The second case is a four-year-old drowning patient with hypoxic-ischemic brain injury. Residents were initially randomly assigned a scenario and asked to break the bad news to the mother, played by a trained standardized patient (SP). Trained SPs were utilized from the standardized patient Program at the University of Texas Southwestern (UTSW) Medical Center. The UTSW Pediatric Emergency Medicine Division provided monetary compensation for the SPs. All simulated encounters were recorded in the ED family consultation room.

Simulation and educational intervention

Residents participated in two randomly assigned simulations: before teaching intervention (pre-test), and one to two weeks post-intervention (post-test). Residents were randomized to either the leukemia or the drowning case. Immediately after the first simulation, the resident was debriefed and given the teaching intervention. The intervention consisted of a 15-20 minutes lecture on BBN, created by the PI in collaboration with the UTSW Palliative Care Department. Content of the lecture included background on BBN, obstacles to delivering bad news, how patients want bad news to be delivered and how to break bad news. One to two weeks later, there is a crossover in the cases. The resident participated in a second case followed by another debriefing session. Three investigators independently scored all pre- and post- test videotaped encounters using the mBAS tool. The investigators were blinded to the order of the videos and to each other’s scores. Investigators were all Pediatric Emergency Medicine attendings and were trained on how to use the mBAS tool.

Data analysis

Data analysis was done using SPSS statistical package software (IBM SPSS Statistics for Windows, Version 22.0, Armonk, NY: IBM Corp; 2013). Descriptive analysis was done to characterize the participants by gender, number of years in training, prior training hours, and residency specialty. All analysis was done separately for each of the five mBAS domains and assumed a p-value <0.05. For each participant, the domain score was calculated by obtaining the mean of the graded scores. The inter-rater reliability was obtained using intraclass correlation (ICC) estimates and their 95% confident intervals based on a mean-rating (k = 3), absolute-agreement and two-way mixed-effects model. An average of the graded scores for the three raters was calculated to obtain the final score. ANOVA F-test was used to determine if the pre-post mean difference significantly differed by participant characteristics. A paired t-test analysis was done to determine if a significant mean difference existed between pre- and post-test scores in each of the five mBAS domains.

## Results

Participation

A total of 35 residents (20 pediatrics, eight emergency medicine, six family medicine, one medicine-pediatric) participated in the course, with one emergency medicine resident unable to finish post-test simulation due to clinical obligations, and therefore was excluded from the study.

Inter-rater reliability

Three raters independently scored both pre- and post-simulation encounters. The ICC values used to evaluate the inter-rater reliability of the average of all raters were 0.41 for Setting the Scene, 0.69 for BBN, 0.40 for Eliciting Concerns, 0.52 for Providing Information, and 0.64 for General Considerations for pre-test scores, indicating a range from substantial correlation (0.61-0.80) to fair correlation (0.21-0.40), according to Landis and Koch’s characterization [24]. The ICC values for post-test score were 0.43 for Setting the Scene, 0.58 for BBN, 0.40 for Eliciting Concerns, 0.60 for Providing Information, and 0.58 for General Considerations, indicating moderate correlation (0.41-0.60) to fair correlation (0.21-0.40) [24]. 

Pre-test and post-test ratings

Pre-test and post-test scores were calculated for each category of the mBAS tool (Table 1). Paired t-test indicated statistically significant reduction (improvement) in mean ratings from pre-test to post-test scores in categories BBN (p= 0.01), Eliciting Concerns (p < 0.01) and Providing Information (p < 0.01). There was also a reduction in pre-test to post-test scores in the General Consideration category (p =0.05), and the Setting the Scene category (p = 0.81); however, results were not statistically significant.

**Table 1:** Paired t-test statistical analysis comparing pre-and post-intervention ratings (n=34)

Domains	Pre-test mean	Post-test mean	Mean Difference(SD)	P-value
A: Setting the Scene	1.93	1.90	0.03 (±0.65)	0.810
B: Breaking Bad News	2.26	1.88	0.38 (±0.81)	0.013
C: Eliciting Concerns	2.56	2.22	0.33 (±0.60)	0.004
D. Providing Information	1.98	1.68	0.30 (±0.61)	0.009
E. General Considerations	1.95	1.73	0.22 (±0.63)	0.054

Co-variables of participants, including gender, number of years in training, prior training, and residency specialty showed no statistical difference in outcomes. Of note, 14 of 34 (41%) of participating residents had no training in communication prior to our study. Nine of 20 (45%) of those with prior training only had one hour of prior education in communication (Table 2).

**Table 2:** Shows the characteristics of participants and the Anova F-test examining the difference in pre- and post-intervention mean ratings by participant characteristics.

Participant Characteristics/ Co-variables	Number (%)	A: Setting the Scene (p-value)	B: Breaking Bad News (p-value)	C: Eliciting Concerns (p-value)	D: Providing Information (p--value)	E: General Consideration (p-value)
Gender		0.254	0.392	0.463	0.106	0.260
Male	13 (38.2)					
Female	21 (61.8)					
Number of years in training		0.654	0.459	0.693	0.392	0.648
One	15 (44.1)					
Two or more	19 (55.9)					
Prior training hours		0.894	0.206	0.600	0.815	0.372
None	14 (41.2)					
One	9 (26.5)					
Two or more	11 (32.4)					
Resident Specialty		0.296	0.04	0.464	0.089	0.232
Emergency Medicine	7 (20.6)					
Family Medicine	6 (17.6)					
Pediatrics/Medicine-pediatrics	21 (61.8)					

## Discussion

This study supports that residents’ BBN skills in the ED improve one to two weeks after a simulation course with educational intervention, suggesting that BBN skills are teachable. In addition, our results demonstrate that there is a lack of communication training for residents and that it is important to improve education on this topic. An increase in communication training using simulation in residency programs can produce significant and meaningful improvements in these skills. 

This is the first study to use an objective tool in assessing BBN performance in the pediatric ED. The improvement in the performance before and after intervention is encouraging. However, this study also demonstrates the need to develop an objective BBN assessment tool for use in the often-chaotic pediatric ED.

Delivering bad news in an emergency setting has its unique challenges, relative to other specialties. The ED is a busy and acute environment, often frightening for patients and parents. Often times, visits represent the first encounter between the patient and the physician, leading to lack of rapport. In addition, ED physicians are often feeling pressured, distracted or preoccupied and juggling a number of patients with life-threatening problems simultaneously. These environmental and emotional factors make BBN even more difficult and delicate in the ED.

While mBAS is an excellent tool for BBN in the Oncology department, its application in the ED may be limited due to the different pace and doctor-patient relationship in the two fields. The raters in our study found the mBAS tool difficult to use since certain questions were not applicable in an emergency setting. For example, question 14 (Appendix 1) asks if the doctor manages to focus on any positive aspect. This may explain the poor inter-rater correlation. Future studies with larger sample sizes are necessary to create an assessment tool specific for BBN in the Pediatric ED.

There were several limitations in our study, including small sample size, single institution, and lack of a control group. There was potential selection bias, given that participants were enrolled on a voluntary basis. Finally, the mBAS tool was created specifically in the oncologic setting, therefore its generalizability in the ED may be limited. 

## Conclusions

Breaking bad news well is an important communication skill every physician should have especially when working in the pediatric ED. This study successfully demonstrates the improvement of BBN performance in residents rotating through the pediatric ED after an educational intervention with simulation. The data support that communication curriculum in residency training programs can improve resident communication skills. This study supports the need for continued focused resident education in communication and BBN.

## Appendices

Appendix 1. Modified Breaking Bad News Assessment Scale (mBAS)

When marking please place a circle around the number which best reflects the score you wish to give. The scale below each question is for guidance only. 

A. Setting the scene: This section looks at whether the doctor established an initial rapport before breaking the bad news. This can be done by creating an environment which allows both private and comfortable communication, by the doctor introducing him/herself, and by the doctor showing an interest in the patient as an individual.

1. How did the doctor arrange the environment?

Very Well                              Very Poorly

       1          2          3          4           5

The doctor may have:

·       Placed the chairs at an angle which allowed unforced eye contact.

·       Ensured that the desk was not in between him/her and the patient.

·       Ensured that the wastebasket was out of the way.

·       Prepared for the patient becoming upset—for example, by placing the tissues so the patient could reach them.

·       Taken measures to prevent interruptions—for example, by disconnecting the phone.

2. Did the doctor appropriately greet the patient and introduce themselves?

Very Well                              Very Poorly

       1          2          3          4           5

The doctor may have:

·       Stood to greet the patient.

·       Confirmed the patient’s name.

·       Introduced him/herself using his/her own name.

·       Given a brief description of his/her occupation.

·       Shown the patient where to sit.

3. Did the doctor show interest in the patient’s current state of well-being and personal circumstances at the beginning of the interview?

Very Well                              Very Poorly

       1          2          3          4           5

The doctor may have:

·       Used open questions

·       Established recent events for the patient.

·       Established the patient’s physical state.

·       Asked how the patient felt emotionally.

·       Inquired into the patients’ social circumstances.

·       Given the patient time to finish their statements. **B. Breaking bad news: This section specifically focuses on whether the doctor was sensitive to the patient’s perspective when he/she delivered the news (the establishment of rapport is scored in the above section). The amount of information given to the individual patient may vary depending on what the patient already knows. Individual patients may differ in the amount of information they wish to receive during this interview, and in the rate at which they assimilate the news.**

4. Before breaking the news did the doctor check what the patient knew already?

Very Well                              Very Poorly

       1          2          3          4           5

Did the doctor:

·       Ask the patient what he/she believed was the nature of their problem?

·       Inquire into what the patient thought the purpose of this meeting was?

·       Check if the patient had thoughts about the possible outcomes from this consultation?

·       Ensure that he/she understood the patient’s perspective at this stage of the interview?

5. Did the doctor introduce the bad news sensitively?

Very Well                              Very Poorly

       1          2          3          4           5

Did the doctor:

·       Gently inform the patient that what followed was going to be important, before using any specific terms?

·       Take cues from the patient on whether to speak or listen after breaking the news?

6. When delivering the news did the doctor utilize an appropriate level of detail and language for the patient’s understanding?

Very Well                              Very Poorly

       1          2          3          4           5

Did the doctor:

·       Begin by using non-specific layman terminology?

·       Respond to the patient’s cues, or ask the patient if he or she wanted more detail, before becoming more specific?

·       Check that the patient was satisfied with his/her own understanding of the terms used?

7. Did the doctor allow the patient to set the pace of the interview?

Very Well                              Very Poorly

       1          2          3          4           5

Did the doctor:

·       Deliver appropriate information when it was asked for?

·       Give the news at a rate which allowed the patient time to think and respond?

·       Check that the patient had understood and assimilated what had been said before giving more information?

8. Did the doctor pause appropriately after giving the news?

Very Well                              Very Poorly

       1          2          3          4           5

Did the doctor:

·       Allow the news about the diagnosis and its implications to sink in?

·       Give the patient time to respond?

·       Appropriately break the silence if the pause was too long?

C. Eliciting concerns: This section focuses on whether the doctor actively attempted to gain a clear idea of the personal implications and meaning of the news to this patient, and the concerns that it generated.

9. Did the doctor specifically ask if the patient had any questions?

Very Well                              Very Poorly

       1          2          3          4           5

The doctor may need to ask for questions repeatedly.

10. Did the doctor explicitly attempt to obtain a complete list of the patient’s concerns?

Very Well                              Very Poorly

       1          2          3          4           5

Did the doctor explore:

·       The patient’s feelings and emotions about the news just given?

·       The patient’s concerns about treatment?

·       The patient’s concerns about prognosis?

·       The concerns arising from family and relationship issues?

·       The patient’s concerns about the effect on their social setting, for example, their employment?

11. Did the doctor explicitly check which areas were most important to the patient?

Very Well                              Very Poorly

       1          2          3          4           5

Did the doctor:

·       Ask the patient which issues were important to talk about during this meeting?

·       Ask in which order the patient wanted to talk about these issues?

D. Providing Information: This section looks at aspects other than giving the news itself.

12. Did the doctor give information tailored to the patient’s expressed concerns?

Very Well                              Very Poorly

       1          2          3          4           5

Did the doctor:

·       Give information in a manner which related to the patient’s expressed concerns?

·       Answer the patient’s questions?

13. Did the doctor clearly explain any information given so that the patient understood?

Very Well                              Very Poorly

       1          2          3          4           5

Did the doctor:

·       Give information in an ordered and logical manner?

·       Use terms appropriate to this patient using plain English and avoiding jargon?

·       Check that the patient understood, and offer clarification?

·       Summarize points for the patient?

14. Did the doctor manage to focus on any positive aspects?

Very Well                              Very Poorly

       1          2          3          4           5

Did the doctor:

·       Frame treatment options in a positive way?

·       Achieve a good balance between explaining benefits and side-effects?

·       Manage to give correct information about the prognosis without extinguishing hope?

15. Was the content of the interview factually accurate?

Always                         Frequently Inaccurate

    1          2          3          4          5

·       If all the information given was factually correct this should gain full marks.

·       If the doctor admitted to uncertainty or lack of knowledge this should still allow full marks.

·       Marks should be deducted for incorrect statements, undue optimism, premature reassurance, or unjustified negativity.

E. General considerations: The following points relate to the interview as a whole.

16. How many of the key areas of the patient’s concerns were touched upon?

All                                           None

  1          2          3          4          5

Each of the following five key areas should be touched upon to obtain full marks:

·       Treatment

·       Prognosis

·       Feelings and emotions

·       Family and relationship issues

·       Effect on social circumstances

17. Were any psychosocial issues exhibited by the patient during the interview explored?

Very Well                              Very Poorly

       1          2          3          4           5

Did the doctor:

·       Acknowledge: the patient’s feelings and emotions; and the effects on family and relationships, and social circumstances?

·       Allow the patient to talk about these issues?

·       Ask questions about them?

·       Enter into a dialogue?

18. Did the doctor manage to appear supportive during the interview?

Very Well                              Very Poorly

       1          2          3          4           5

Did the doctor:

·       Show warmth?

·       Show emotional support?

·       Convey a sense that this really mattered to the doctor?

·       Convey a personal sense of strength and resourcefulness that was available to help the patient?

19. Did the doctor use appropriate body language during the interview?

Definitely                                  Not at all

       1          2          3          4           5

Did the doctor:

·       Maintain an appropriate level of eye contact?

·       Look interested and alert to the patient’s needs?

·       Show a competent and caring professional manner?

20. Did the doctor avoid appearing clumsy during the interview?

Very Well                              Very Poorly

       1          2          3          4           5

Did the doctor:

·       Introduce difficult topics gently?

·       Deal with painful issues sensitively?

·       Show flexibility and sensitivity to the patient’s needs?

·       Avoid non-sequitur?

·       Avoid using inappropriate phrases?

21. Did the doctor tailor the pace of the interview to suit the patient?

Very Well                              Very Poorly

       1          2          3          4           5

Did the doctor:

·       Let the patient speak without interruption?

·       Respond to the patient’s cues regarding timing and delivery?

·       Deliver appropriate information when it was asked for?

·       Use pauses where appropriate to give the patient time to think and respond?

·       Check that the patient had finished with a topic before moving on to another?

22. Did the doctor manage the time available?

Very Well                              Very Poorly

       1          2          3          4           5

Did the doctor:

·       Sensitively make the patient aware of how much time was available for discussion?

·       Mention the opportunity of further interviews to the patient?

·       Cover the important issues in this session?

·       Make a plan for future action?

·       Bring the interview to a conclusion?

Appendix 2: Resident survey

1.    What is your residency specialty?

a.    Pediatrics

b.    Emergency medicine

c.    Family medicine

2.    What year are you in your training?

a.    PGY-1

b.    PGY-2

c.    PGY-3

3.    Have you had prior breaking bad news training? If yes, how many hours total? If yes, please describe the training. 

a.    Yes. Number of hours ____. 

                  i.     Description of prior training:

b.    No

4.    What is your gender?

a.    Male

b.    Female 
